# Mentalizing Imagery Therapy Mobile App to Enhance the Mood of Family Dementia Caregivers: Feasibility and Limited Efficacy Testing

**DOI:** 10.2196/12850

**Published:** 2019-03-21

**Authors:** Abu Taher Sikder, Francis Cheng Yang, Rhiana Schafer, Glenna A Dowling, Lara Traeger, Felipe Ananda Jain

**Affiliations:** 1 University of California, Berkeley Berkeley, CA United States; 2 Department of Neurology Feinberg School of Medicine Northwestern University Evanston, IL United States; 3 Department of Physiological Nursing University of California, San Francisco San Francisco, CA United States; 4 Department of Psychiatry Harvard Medical School Boston, MA United States; 5 Depression Clinical and Research Program Department of Psychiatry, Massachusetts General Hospital Harvard Medical School Boston, MA United States

**Keywords:** family caregivers, mindfulness, depression, mobile apps, psychotherapy

## Abstract

**Background:**

Family caregivers of patients with Alzheimer disease and related dementias (AD and ADRD) often experience high stress and are at high risk for depression. Technologically delivered therapy is attractive for AD and ADRD caregivers because of the time demands associated with in-person participation.

**Objective:**

We aimed to study the feasibility and conduct limited efficacy testing of a mobile app intervention delivering mentalizing imagery therapy (MIT) for family caregivers.

**Methods:**

A 4-week trial of the MIT app for family AD and ADRD caregivers was conducted to assess the feasibility of use and investigate changes in depression symptoms, mood, and caregiving experience. Semistructured interviews were conducted to characterize participants’ perceived feasibility and benefits.

**Results:**

A total of 17 of the 21 (80%) consented participants (mean age 67 years, range 54-79) utilized the app at least once and were further analyzed. Average usage of audio recordings was on 14 (SD 10) days out of 28 possible and comprised 29 (SD 28) individual sessions. There were improvements in depression with a large effect size for those who used the app at least moderately (*P*=.008), increases in positive mood postintervention (*P*<.05), and acute increases in mood following daily guided imagery practice (Stretching and Breathing, *P*<.001; Eye in the Center, *P*<.001; Nesting Doll, *P*=.002; Situation Solver, *P*=.003; and Life Globe, *P*=.006). Semistructured interviews revealed perceived benefits such as greater ability to remain “centered” despite caregiving challenges and positive reframing of the caregiver experience.

**Conclusions:**

App delivery of MIT is feasible for family AD and ADRD caregivers, including aging seniors. Results showed moderate to high usage of the app for a majority of users. Limited efficacy testing provides justification for studying the MIT app for AD and ADRD caregivers to improve mood and reduce depression in larger, controlled trials.

## Introduction

### Background

Despite frequently bearing significant physical, socioeconomic, and psychological burdens, millions of family members persist in providing informal care for a loved one with dementia [1]. In 2015 alone, it was estimated that informal caregivers of patients with Alzheimer disease and related disorders (AD and ADRD), the vast majority of whom are family members, provided 18.1 billion hours of unpaid assistance, saving the health care system more than US $200 billion a year [2]. The stresses of caregiving for a loved one with memory loss and behavioral changes frequently include sleep loss and other physically strenuous tasks such as assisting with bathing or dressing, role conflict with work and other interests, financial difficulties, and grief related to deterioration of the loved one and losses in the relationship [1-4]. Assuming the role of family AD and ADRD caregiver has been associated with a higher risk of depression and anxiety [5,6], compromised immune function [7], and increased mortality [8,9].

Several studies and systematic meta-analyses have found that mindfulness and related guided imagery interventions, which have salutary effects on depression and anxiety [10,11], hold promise for improving the health of family AD and ADRD caregivers by reducing negative psychological symptoms and increasing quality of life [12]. For example, caregivers undergoing mindfulness-based stress reduction reported lower depression and stress after the intervention [13,14]. A yogic meditation and imagery technique improved the mental and cognitive function of family dementia caregivers [15]. As the stressors that caregivers face are relational and not solely individual, treatments for caregivers that incorporate exercises that target reducing stress and address interpersonal challenges may be helpful. Our work with a relational guided imagery approach that incorporates principles of mentalization with mindfulness demonstrated benefit for insomnia, depression symptoms, and anxiety in an in-person pilot 8-week trial [16].

These promising data for alleviating symptoms of family AD and ADRD caregiver stress and depression required caregivers to come to the study site to receive in-person treatment and, thus, limited the pool of caregivers to those in local catchment areas who lived near academic medical centers. The dissemination of in-person interventions such as these is often limited by time to travel to the clinic and receive in-person care, transportation constraints, and, particularly in rural areas, a scarcity of trained professionals to provide high-quality specialty care [17,18]. The presence of these barriers means that, although mindfulness and guided imagery may help alleviate caregiver depression and anxiety, the interventions remain inaccessible for a sizeable number of caregivers.

Mobile apps are software programs residing on a portable device such as a smartphone, watch, or tablet. Advantages of app technology for therapy delivery include its ease of access to information, capability to deliver home practice exercises such as audio recordings, ability to send notifications and reminders, and potential to capture active and passive usage feedback. Apps may house information available for offline use, connect to websites, or both. Apps with information available offline may be ideally suited for delivery of therapies that provide regular home practice exercises so that caregivers do not need to connect to the internet each time they wish to use them.

Several apps have been targeted to ameliorate depression symptoms, by providing cognitive behavioral therapy [18,19] and behavioral activation [20], but none of which we are aware specifically promote a balanced understanding of the mental lives of oneself and others. We are aware of only 2 published studies using an app to deliver interventions to informal caregivers: a study of psychoeducation, in which the app platform was not reported to be a feasible delivery method [21], and a small open-label feasibility study to help share information among caregivers, case managers, and physicians [22]. Both of these studies used the app interface to connect to content hosted on the internet, thus maximizing connectivity but limiting accessibility to times when caregivers were online.

### Objectives

To our knowledge, apps that deliver mindfulness or guided imagery practices to family dementia caregivers have not been studied. We investigated the feasibility for caregivers of a mobile technological app to deliver mentalizing imagery therapy (MIT), which incorporates guided imagery and mindfulness to facilitate self-regulation and increased perspective taking on the mental life of self and others [23]. Relative to other mindfulness techniques and apps of which we are aware, MIT occupies a niche in directly targeting the self and other understanding necessary to navigate challenging relationships when interpersonal understanding (mentalizing) breaks down [23]. Given prior preliminary successes of in-person groups using MIT techniques in reducing depressive symptoms of dementia caregivers [16,24] and the accessibility provided by smartphone apps, we hypothesized that this novel integration would be a feasible method of treatment for dementia caregivers. Consistent with the goals of a stage I feasibility study [25,26], we also aimed to conduct limited efficacy testing over the short term (4 weeks) and hypothesized that caregivers using the MIT app would experience an improvement in mood, reduction in depression, and benefits for the caregiving experience.

## Methods

### Overview

A 4-week open-label trial was conducted to test the feasibility of a remote MIT app in 21 family dementia caregivers. Participants were recruited with Facebook advertisements and flyers posted on the internet, provided at Alzheimer Association community meetings, and sent to the known dementia caregiver pool at the University of California, San Francisco (UCSF). The MIT app was approved by Apple and hosted on the app store [27] for participants to download onto a compatible iOS (version 9.3+) device such as iPhone, iPad, or iPod Touch. All procedures involving human participants were approved by the Committee for Human Research at UCSF.

### Participants

**Figure 1:**
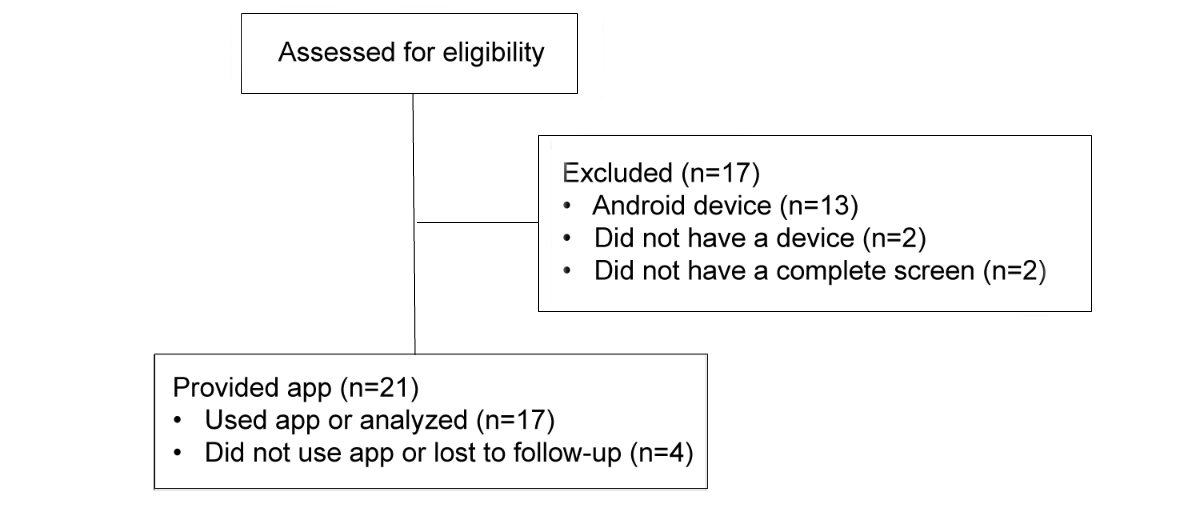
Consolidated Standards of Reporting Trials flow diagram.

Participants were eligible for the study if they were (1) English speakers, (2) 45 years or older, (3) had access to an iOS smartphone or compatible device, and (4) reported being the primary caregiver for a relative with dementia. Participants were excluded if they had active suicidal ideation or thoughts of violence toward others. The Consolidated Standards of Reporting Trials flow diagram for the study is presented in Figure 1.

### Procedure

Participants underwent a 15-min telephone screening interview by a trained research assistant. If participants endorsed thoughts of suicide or violence, a comprehensive risk assessment was performed over the phone by a faculty psychiatrist. Written informed consent for participation was obtained by email. Following informed consent, participants completed online questionnaires measuring depression and mood using REDCap (Vanderbilt University). They were then directed to download the app from the Apple store and provided a personalized activation code. An optional conference call was offered for questions regarding download or activation. After 4 weeks of app usage, participants were sent the same set of online questionnaires, and a semistructured interview was performed with the first 8 completers by a fourth-year doctoral student in clinical psychology.

### Intervention

MIT is a guided imagery and mindfulness intervention that incorporates principles of mentalization [23]. Mentalization refers to the process by which we consider and understand mental states along different dimensions: self and other, cognitive and affective, implicit automatic cognition and explicitly controlled, direct internal consideration of thoughts and feelings, and observation of external facial and behavioral cues [28]. MIT endeavors to help participants find balance among these different poles of mentalization. MIT also incorporates exercises to help participants mindfully observe themselves and understand the interconnectedness of self and other. The MIT app consisted of audio recordings of the MIT practices and 4 essays (1 for each week) explaining the concepts underlying each recording and supportive information, including specific stories of how family dementia caregivers used the techniques (Table 1). In addition, in the written instructions accompanying each week, participants were advised to listen to the audio recordings twice a day in the first week and once a day thereafter.

### Measures

In-app data related to usage of specific audio recordings or reading material were obtained passively, and participant ratings were actively collected. These data were downloaded live from the device (or at the next online connection if the device was not connected to the internet during use) and saved to a Mixpanel database [29]. Depression and mood self-reports were obtained at baseline and after week 4 with REDCap-delivered assessments. In addition, they were obtained in the app after weeks 1, 2, and 3.

*Depression* was measured with the 16-item Quick Inventory of Depressive Symptoms–Self-Rated (QIDS), and severe depression was identified using a validated cutoff of 16 on the QIDS [30]. *Mood* was assessed with the Positive and Negative Affect Scale [31].

### Acute Mood Change and Attention

During-meditation acute mood change and attention were obtained by self-report in the MIT app. After participants listened to an audio recording, they were prompted to rate their experience on a screen that included 3 Likert scales and a slider to select a numeric value. The first 2 scales measured “Overall feelings before practice” and “Overall feelings after practice” with a scale bounded by −5 to 5 and with intermediate integer values. Above −5 was a sad emoji, above 0 was a neutral emoji, and above 5 was a smiling emoji. The third app scale measured attention using the question: “How well did you focus,” with a similar scale of −5 to 5. Above −5 was *None*, above 0 was 50%, and above 5 was 100%.

### Helpfulness of Reading

After visiting a page with reading material, the subject was prompted to rate the helpfulness of the material with a single item, “How helpful was this reading” on a scale of −5 to 5 with emojis identical to those for the immediate mood ratings.

**Table 1:** Mentalizing imagery therapy experimental mobile app content and modules.

Module		Content
**Imagery and mindfulness audio**		
	Stretching and Breathing	Guides the subject through a series of stretching exercises and a mindful breathing exercise.
	Eye in the Center	Instructs users to focus on their sensations, thoughts, and feelings *mapped out* relative to their center (defined as a region in the lower chest midway between the base of the spine and the top of the head).
	Nesting Doll	Instructs users to bring an image (visual or nonvisual) in the center region that includes a felt sense of their moment-by-moment internal thoughts, feelings, and sensations. The meditation progresses to imagine a nesting doll of a loved one (usually the dementia patient) along with the imagined loved ones’ thoughts and feelings. Participants may send themselves and the loved one positive wishes “if they feel ready.”
	Situation Solver	Guided imagery that involves recalling a challenging experience from the perspective of self and loved one. Uses imagery rehearsal to reimagine the situation according to their values.
	Life Globe	Meditation that involves becoming aware of one’s connectedness with others, communities, the earth, and the larger universe. Takes perspective of the larger whole at each stage.
**Additional features**		
	Mindfulness Tool	Mnemonic *BAM* (Breathing, Awareness, and Motivation) provided to help the subject quickly take steps to destress by focusing on breathing, awareness, and motivation (intention behind their actions).
	Daily Reminders	Two daily notifications in the morning and at night were sent to remind the user to take time for themselves and meditate, practice an imagery technique, or use other features of the app. If the app was used in the morning, the night notification would not be triggered.

### Exit Interview

Individual interviews based on a semistructured interview guide were conducted by videoconference with the first 8 participants who completed all 4 weeks of the intervention to understand the caregivers’ perceived utility of the app in terms of their well-being, relationships, and sense of connectedness to others. Participants were asked questions such as “How did the app impact you?,” “Do you think that the app changed your relationship or sense of connectedness with others?,” and “Do you have any suggestions for improvement regarding the application?”. Specific components (MIT exercises and readings) were reviewed, and subjects were asked whether each element was “helpful, and if so, how?”. Broad suggestions were solicited regarding any improvements to the experience of using the app. Narratives were transcribed and responses were categorized into whether they indicated positive or negative experiences, difficulties, self-understanding, connectedness to others, mindfulness, and comments related to specific MIT components. Responses were then reviewed for perceived helpfulness, negative experiences or difficulties, types of benefits elicited by each MIT exercise, and suggestions for improvement of MIT app delivery.

### Statistical Analyses

Outcome analyses were performed on the sample of users who downloaded and used the app at least once. Analysis of mood trajectory was performed with R 3.3.2 [32]. All data were tested with the Shapiro-Wilke test and found to not deviate from normality. Linear mixed models were used to assess depression and affect trajectory with time as the independent variable, adjusting for age and sex, and subject as a random factor using lmer in package lme4. *P* values were estimated using the Wald chi-square test (function analysis of variance in library car). Depression symptom trajectory was also assessed in the overall sample and in the subset of participants reporting severe depression symptoms. Usage was calculated as the total number of audio practice sessions the participants completed. Predictive analyses were performed to assess the impact of age, sex, or baseline mood on usage with linear models across all participants who completed the baseline screening assessments. Paired, 2-sided *t* tests were performed on (1) Likert mood ratings from before to after audio-guided practice sessions across participants and (2) QIDS ratings at baseline and last available QIDS completed. Cohen *d* for change in depression symptoms was calculated as the mean of the change in QIDS divided by SD of the change. The relationship between attention during a practice session and immediate mood change was evaluated by pooling data from across all participants and meditations and using a linear mixed-effects model with subject as a random factor, rating of focus as the independent variable, and immediate change in mood (mood after meditation−mood before meditation) as the dependent variable.

## Results

### Participants and Demographics

Figure 1 illustrates the flow of participants through the trial. Out of the 17 participants included in the analysis, 71% (or 12/17) were women and age was 66.52 (SD 8.61) years. All participants were white.

### Feasibility

#### Imagery and Mindfulness Practice Data

##### Audio Recording Usage

Usage frequency was conservatively estimated in participants who listened to an audio recording at least once and also rated its effect (n=17 participants). Thus, if participants listened to part of a recording (or even the whole recording) but did not rate their mood change, this usage was not captured. Average usage across these 17 participants was on 14 (SD 10) days out of the 28 days possible, and 29 (SD 28) individual audio sessions were conducted. Examination of usage revealed that participants fell into 1 of the 3 distinct patterns that were almost equally split among the participants: low, or usage on less than 1/3 of days (n=6 participants); moderate, or usage on 1/3 to 2/3 of days (n=6 participants); and high, usage on more than 2/3 of days (n=5 participants). Most low users, 6 out of 17 participants (35%), tried the app a few times and then discontinued use; the average number of audio sessions was 5 (SD 6) over the 28 days. The moderate group contained a mix of participants whose use declined and also some users whose use increased; the average number of audio sessions was 25 (SD 6). The high-use group typically listened to an audio recording twice a day (and sometimes more); the average number of sessions was 64 (SD 24). Total usage among all 21 participants who were provided the app was not predicted by sex, age, or baseline depression symptoms, nor was usage among the 17 participants who used the app at least once *(P*>.3 for all comparisons).

##### Acute Mood Changes

Immediately after completing an exercise, participants rated their mood before and after the meditations. For each type of practice, average changes (on the 11-point Likert scale) in acute mood across participants were in the direction of improvement as follows: Stretching and Breathing 1.65 (SD 1.39) points, *t*_15_=4.72, *P*<.001; Eye in the Center 2.17 (SD 1.67), *t*_16_=5.36, *P*<.001; Nesting Doll 1.81 (SD 1.56), *t*_11_=4.02, *P*=.002; Situation Solver 1.88 (SD 1.54), *t*_9_=3.86, *P*=.003; and Life Globe 0.81 (SD 0.61), *t*_7_=3.78, *P*=.006.

##### Stability of Focus

Across meditations, the average stability of focus was 2.14 (SD 1.81), corresponding to paying attention about 70% of the time. Greater focus during a meditation session was highly predictive of improvement in immediate mood (Wald χ^2^_1_=47.0; *P*<.001) across participants.

#### Knowledge Data Usage and Helpfulness

For the 4 informational documents in the app, subject’s reading usage was estimated based on the number of times they read the material and also rated its helpfulness. Out of the 17 users, 3 (18%) read all 4, 6 (35%) read 3, 3 (18%) read 2, and 5 (29%) read 1. Average *helpfulness* was 2.87 (SD 1.11) on the Likert scale (from −5 to + 5) or about 81% of the distance toward the upper bound.

#### Symptom Changes: Negative Affect, Positive Affect, and Depression

Linear mixed effects models in the 17 participants who used the app at least once were used to determine changes in affect over time, adjusting for sex and age. Negative affect decreased irrespective of age or sex: time χ^2^_1_=10.2, *P*=.001; age χ^2^_1_=0.2, *P*=.70; and sex χ^2^_1_=0.4, *P*=.53. In the model predicting positive affect, both time and age were significantly predictive of increased positive affect over the course of the study: time χ^2^_1_=4.5, *P*=.035; age χ^2^_1_=11.4, *P*<.001; and sex χ^2^_1_=0.1, *P*=.77. There was a trend toward improvement of depression symptoms across all participants: time χ^2^_1_=2.3, *P*=.13; age χ^2^_1_=3.4, *P*=.065; and sex χ^2^_1_=0.8, *P*=.38. Those subjects who used the app at least moderately (n=11) evinced a significant drop in average QIDS with a large effect size, from 9.72 (SD 4.38) to 7.82 (SD 3.68), *t*_10_=3.30, *P*=.008, Cohen *d*=.99. Subjects not using the app regularly (n=5 completing QIDS) showed no change in average QIDS, 12.4 (SD 4.72) to 14.2 (SD 11.73), *t*_4_=.33, *P*=.75, Cohen *d*=.15. In the 3 participants with severe depression (QIDS≥16 at baseline), depression significantly decreased over time: time χ^2^_1_=7.2, *P*=.007; age χ^2^_1_=0.3, *P*=.57; and sex χ^2^_1_=1.4, *P*=.24.

#### Exit Interview

Caregivers commonly described benefits of the app to their emotional and cognitive well-being, such as feeling more “centered,” “anchored,” “on an even keel,” and learning to “not get too overwhelmed” by their emotions. Caregivers found that these changes helped them to better care for their loved ones or accomplish what they needed to do, or with one caregiver feeling more like his or her “normal self.” About half of the caregivers also reported improvements in perspective taking with their care recipient and the disease process, for example, “realizing that when I’m mad at her it’s actually I’m mad at the disease she has.” Several caregivers reported that the app helped them shift their experience of caregiving, learning that “the person you’re caring for can be an inspiration and the light in you” and finding “a more positive way of focusing on the negative things that have happened.”

Overall, 7 of the 8 participants felt moved by at least one of the audio recordings and expressed preference for some recordings over others. Specific feedback on patient preferences supported that the individual techniques may have yielded intended effects. For instance, exercises focused on self-regulation (Breathing and Stretching and Eye in the Center) were experienced as soothing and centering, whereas the Nesting Doll and Situation Solver perspective-taking exercises that focused on mentalizing the loved one resulted in greater perspective taking. The Life Globe meditation, which taught connectedness with others and the world, was experienced as “really very comforting” or that it left the caregiver with an “expanded viewpoint.” In comparison, a minority of subjects found the mindfulness or internally focused attention exercises to be “disorienting” or found the imagery exercises to be too complex. Uniquely, 1 individual who reported that mindfulness had “always been a challenge” reported no benefit from the meditations but found the readings to be “phenomenally” helpful.

Participants also described some caregiver-specific challenges in engaging with the app, such as pulled away from the app by the care recipient and being asked to focus on a specific challenging caregiving experience, which for some was emotionally arousing. None of the caregivers reported technical difficulties with the app interface, and a few caregivers commented that it was “user friendly” and several mentioned that the notifications provided “a good reminder” to use the app. A few participants commented that they would have benefited from contact with an interventionist for support and guidance to ensure they were “on the right track.”

## Discussion

### Principal Findings

The MIT app demonstrated promise in 4 main areas: technical feasibility, usage, mood improvement, and relational understanding. The large majority of caregivers receiving the app downloaded it and listened to at least one audio recording. A few of the participants mentioned that the app was easy to use or improved convenience, whereas none mentioned technical difficulties as being a barrier to app use, suggesting that the design interface was accessible to this elderly cohort. This is the first demonstration that an entirely remote mobile app technology delivering MIT, without ongoing therapist guidance or content instruction, may be successfully delivered to an aging cohort of participants including seniors.

Download rates and usage of the MIT app were variable but overall higher than what we expected based on other reports of download and usage of psychotherapy apps in adults with low mood [18] or of mindfulness apps to reduce stress and improve mental health in general populations [33,34], in which participants downloaded or used the app at about a third to a half the frequency of what we observed. Reasons for this are unclear but could be because of our participants being caregivers, who overall are quite conscientious and highly motivated, or other differences in study design related to app content, participant selection, or the nonrandomized nature of our trial.

In our population, usage patterns varied by participant and appeared to fall into distinct groups. Two-thirds of participants fell into a moderate- or high-usage group, suggesting feasibility for these participants. Strikingly, about a third of participants used the app on average more than once a day, suggesting that for a sizable minority of caregivers, guided imagery and meditation exercises delivered via the app can become incorporated routinely at least over the 4-week period we studied. However, for one-third of the participants who tried the app, usage was low, suggesting a lack of feasibility for a subset of participants. Features at baseline such as depression symptoms, age, and gender did not predict this difference in usage; further research may be beneficial with qualitative methods to identify reasons for low usage.

There were acute effects on improving mood associated with practicing the guided imagery and mindfulness exercises, and there were significant findings at 4 weeks for improving overall positive mood and reducing negative mood. Complementing the quantitative findings were caregivers’ perceived benefits that the app provided “calm,” “lowered stress,” and provided an “anchor” for their mood. These findings add to the body of evidence that mindfulness app use in adults [33,34] and technological therapy delivery for caregivers [35,36] improves symptoms. Reassuringly, for the most severely depressed participants in our population, there was no worsening over the 4 weeks, and on the contrary, there were promising signs for reducing depressive symptoms. The magnitude of depression symptom reduction was approximately 30%, indicating that the MIT app alone is not likely to be sufficient for fully resolving depression symptoms, but it might be beneficial to study for depression in combination with other treatments, as an augmentation therapy, or with the guidance of a therapist.

Caregivers’ perceived benefits for relationships and connectedness took different forms. Participants found themselves better able to take the perspective on their loved one and recognize the role that the illness was playing in dementia. This suggests that the app helped to enhance mentalizing capacity in relation to their loved one. For some caregivers, a complex reframing of the caregiver experience resulted, such as being able to see their loved one as a “light and the inspiration” inside of them after the Nesting Doll. The Life Globe exercise particularly resulted in finding “comfort” in connectedness, and by recontextualizing themselves as part of a larger whole, discovering “a more positive way of looking at the negative.” Remarkably, this higher-order reframing of themselves and their loved ones took place without therapist contact but merely by interacting with the app components. Further studies should address whether improved perspective taking and reframing may be mediators of effects on mood. The relational impact of the MIT app also provides support for studying it for other populations with high levels of interpersonal stress, who might specifically benefit from the balanced attention to self and others provided by the MIT exercises.

Regarding specific app components, individual caregivers clearly exhibited preferences for particular exercises. Most caregivers found the guided imagery and mindfulness exercises helpful. The subject who uniquely reported that none of the mindfulness or guided imagery exercises helped still found value in the reading material. Thus, providing a menu of options from which caregivers could choose increased the benefit of the app for a broader range of caregivers. Future studies targeted at identifying active components of the MIT exercises would thus need to account for moderating factors leading to individual preferences and also clearly specify which therapeutic goals of MIT were being examined (eg, affective self-regulation, mentalization of self, and mentalization of others).

### Limitations

Limitations of the study include the small sample size for our statistical models, which could have predisposed to type II errors; lack of a control group to account for nonspecific effects of being in the trial; and low ethnic and racial diversity of participants. As other studies have found that minorities will engage in mobile app therapies [37] and have previously reported benefit with in-person MIT delivery [16,24], improving recruitment methods to target these populations online will be important in future studies. The interviews suggested that people having a history of feeling challenged by mindfulness practices may need more guidance. Regular contact with an instructor for encouragement, identification of experience, explanation of some of the more difficult exercises, and support might help. In addition, an interventionist might help participants engage in problem solving regarding a place and time to do the meditation without interruption by their loved ones. Moreover, prior familiarity with mindfulness and guided imagery techniques and with smartphone use was not assessed, and these would be helpful to study as potential moderators in future trials. Furthermore, information regarding concurrent depression treatments was not collected, and this would be helpful for determining the specificity of effects and how caregivers used the MIT app (ie, as a solo therapy or as augmentation of other mental health interventions). Although overall feedback was positive regarding the app design, research with quantitative, empirically based tools may help to more rigorously characterize usability.

### Conclusions

Our findings demonstrate that using the MIT app was feasible for the majority of family caregivers who enrolled in the trial. The MIT app showed promising results in positive affect increase, decrease in negative affect, and depression improvements for participants reporting high depression symptoms. Moreover, reports were consistent with increased mentalizing of the care recipient and higher-level reappraisal of the caregiver experience. These findings provide justification for larger, randomized controlled trials that could address specificity of MIT app benefits for family AD and ADRD caregivers.

## Abbreviations

AD: Alzheimer disease
ADRD: Alzheimer disease and related dementias
BAM: breathing, awareness, and motivation
MIT: mentalizing imagery therapy
QIDS: Quick Inventory of Depressive Symptoms–Self-Rated
